# A novel approach for minimising anti-aliasing effects in EEG data acquisition

**DOI:** 10.1515/biol-2022-0664

**Published:** 2023-10-03

**Authors:** Putta Brundavani, Dupakuntla Vishnu Vardhan

**Affiliations:** Department of ECE, JNTUA, Ananthapuramu, A.P., 515 002, India

**Keywords:** electroencephalography, data acquisition system, anti-aliasing filter, analogue-to-digital converter, signal-to-noise ratio, successive approximation

## Abstract

Electroencephalography (EEG) waves and other biological signals can be deciphered with a deeper understanding of the human body. The benefits of EEG are growing. EEG studies have expanded globally. Research on EEG covers data gathering, analysis, energy renewal, and more. EEG-gathering devices include encoding, digital transfer, head sensor placement, and separate amplifiers. The EEG detects periodic noise. Head movement, sensor lines, and hair sweat produce low-frequency noise. Low-frequency noise alters EEG signals over time. Muscle actions and electromagnetic waves create high-frequency noise (especially in the facial and neck muscles). EEG shifts are saw-toothed by high-frequency noise. High- and low-frequency noises are usually lower and higher than human EEG, respectively. Lowering signal power above and below the testing level without altering the signs of interest lowers noise. Aliasing may affect low-frequency impacts in the original data because high-frequency noise is mirrored in the data. This work designed a non-binary Complementary metal oxide semiconductor (CMOS) Consecutive guesstimate register (CGR) reconfigurable analogue-to-digital converter (ADC) integrated with the instrumental amplifier. CGR ADC model comprises the bio-signal device monitoring for the EEG signals. This study focused on acquiring the EEG signals for amplification. The model uses the AC-coupled chopper stabilisation model with 1 A low power with a noise level of 1 A. The neural amplifier uses an optimised current technique to maximise the transconductance for a good noise efficiency factor. The simulation analysis estimates a bandwidth range of 0.05–120 Hz with a power consumption level of 0.271 µW. The computed noise level is observed as 1.1 µV_rms_ and a gain of 45 dB. The comparative analysis of the proposed ADC model achieves the minimal energy consumption value of ∼12%, which is minimal than the nonlinear and switch-end capacitor. Also, the time consumed is ∼9% less than the nonlinear and switch-end Capacitor.18 nm CMOS technology is used to implement the proposed data acquisition system for low-power and density-optimised applications.

## Graphical abstract

**Figure:**
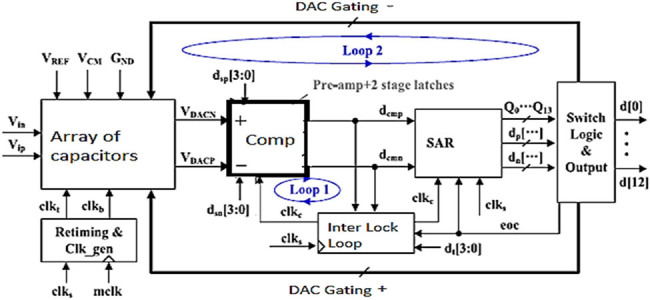

## 1 Introduction

People’s aspirations for a stress-free and hassle-free existence are evolving alongside the rise of modern technology. The health of humans is monitored by paying heed to biological signals. Therefore, studying human biochemical signals is growing in significance. Physiological data such as heart rate, respiration rate, blood pressure, and pulse are all examples of typical signals. Since electroencephalogram (EEG) data originate in the brain’s crucial processes, they have received more attention.

Furthermore, scientists can use the data to learn how the brain operates. The EEG is a microvolt-level magnitude time-varying non-stationary signal. Frequency-domain and time-domain analysis, wavelet transform, wavelet entropy, improved multi-scale entropy algorithm, and EEG feature extraction based on a constrained independent component are popular approaches for analysing and processing EEG signals. EEG is currently used in the medical field but will expand into new areas, such as the military, athletics, educational psychology, entertainment industry, etc. Moreover, the application of EEG in various domains will profoundly impact our civilization.

Humans have maintained their EEG research long after Adolf Beck’s first studies. The first human EEG, recorded by Hans Berger, represents a watershed moment in the history of clinical neuroscience. The interictal surge was the first thing that Gibbs and Jasper noticed as different about people with epilepsy. After that, EEG was first used in clinical practice. The development of the brain–computer interface (BCI) made it possible for the first time for people to use their thoughts to direct real objects through a labyrinth. In addition, tetraplegic Matt Nagle was the first to control an artificial hand via a BCI. Researchers are getting closer to developing “Wearable EEG” technology by decreasing the size of EEG equipment.

The BCI and illness diagnosis might both gain from this development. Modern EEG and machine learning can automatically assess data, leading to more accurate illness detection. Recently, the advancement of the analogue world with digitisation exhibits technological advancement with the implementation of the Internet of Things, big data, and cloud [1]. The data acquisition device incorporates an analog-to-digital converter (ADC) to increase the big data accuracy. The advancement in the design of ADC focused on precision digital designing throughout the applications [2,3,4,5,6]. Through historical metrology, the speed and resolution are increased within the ADC devices [7]. Different applications require high performance to achieve desired ADC in different areas [8]. Examples of ADC are program controllers, process control, control of the electric motor, and electrical energy distribution. With minimal instrumentation, domain testing, development, research, and qualification are adopted to achieve higher precision in the conversion of digital transformation [9]. At present, a vast range of ADC architectures are implemented to increase the precision aligned with the principle of ADC. The example of those is successive approximation register (SAR). The design of SAR comprises 24 bits resolution with minimal MSPS and 32 bits of several hundred Kbps [10]. Conventionally, the ADC belongs to the class of analogue signals provided with a fixed frequency range [11].

The uniform time grid consequences provide the sampling instants denoted as the spectral aliasing [12]. Spectral aliasing is shown by the frequency components that are higher than the Nyquist frequency and are folded back into the Nyquist zone [13]. The wireless communication comprises the multi-stand and multi-band operations for the tunable analogue filters or switched filter bands that provide the effective antialiasing filtering with the uniform sampling of Nyquist ADC conversion to eliminate the out-of-band signal process. The sampling scheme is also stated as non-uniform sampling to achieve effective cross-level quantisation [14]. To eliminate anti-aliasing effects in EEG signal data collection, this work obtained a Consecutive guesstimate Register (CGR) ADC design. Digital low-pass filters (LPFs), A/D converters, and compensators make up the model. The suggested model’s performance is assessed with 3 and 5 V input voltage signals. Comparative investigation showed that the suggested ADC model consumes 12% less energy than the nonlinear and switch-end capacitor. The time is ∼9% less than the nonlinear and switch-end capacitor.

The EEG picks up periodic noise. Head movement, sensor lines, and hair sweat make low-frequency noise. Low-frequency noise changes EEG signals over time. High-frequency noise is made by muscle actions and electromagnetic waves, especially in the facial and neck muscles. High-frequency noise makes EEG shifts look like a sawtooth. High-frequency noise is usually lower than human EEG, and low-frequency noise is usually higher.

### 1.1 Contribution to the work

- This article proposed a non-binary Complementary metal oxide semiconductor (CMOS) CGR reconfigurable ADC combined with the instrumental amplifier. CGR ADC model monitors EEG biosignals.
- The proposed technique is centred on capturing EEG data for the amplification procedure and employs a low-power, high-noise version of the AC-coupled chopper stabilisation scheme.
- The optimised current technique of the neural amplifier helps it attain high transconductance and low-noise efficiency.
- The proposed model simulation analysis estimates a bandwidth range of 0.05–120 Hz with a power consumption level of 0.271 µW. The computed noise level is observed as 1.1 µV_rms_ and a gain of 45 dB.
- The automated model shows less power consumption by the proposed reconfigurable ADC in the front-end devices. The data-acquisition circuitry is used in reconfigurable ADCs. Based on the input amplitude and resolution, the circuit works well for the specified configuration at the sample rate of the input signal frequency.
- The suggested ADC model is 12% more efficient in energy usage than the nonlinear and switch-end capacitors. Compared to the nonlinear and switch-end capacitors, the time required is also significantly reduced by 9%. The suggested data collection system uses 18 nm CMOS technology, allowing low power consumption and high density.

The remaining sections of the study are organised as follows. Recent research on the proposed method is discussed in Section 2. Section 3 defines the proposed method. Section 4 shows the simulation results of the proposed method. Finally, the Conclusion is described in Section 5.

## 2 Related works

Using the ADC front-end ADS1299FE, an FPGA Zedboard (Diligent, USA)-based EEG data acquisition device has been built. The voltage potential of an EEG signal is typically between 0.5 and 100 V, and its frequency is typically between 0.5 and 40 Hz, both of which are measured using an EEG instrument placed on the head. The 2-chip ADS1299 Daisy-Chain mode and Zedboard FPGA-based computing comprise the data acquisition system’s configuration. Because of its amazing speed at which it can collect data from the ADC devices, FPGA was included in the data acquisition system [15]. The EEG data have been transferred from the data acquisition device to a personal computer and displayed graphically. The EEG device has been verified with an EEG Simulator (NETECH Mini-Sim EEG) at 2 Hz, 5 Hz, 30 µV, 50 µV, and 100 µV for voltage. The 2 Hz validation result was measured with a maximum average variation of 0.15 µV, and the 5 Hz result was measured with a maximum average deviation of 0.18 µV.

Research into the human body continues to advance, and with it comes a new understanding of human biological signs, most notably the EEG signal. The advantages of EEG are also becoming increasingly well-known. As a result, interest in EEG studies has increased globally. Research into EEG is currently being conducted in many areas and phases, including data acquisition, data analysis, energy recovery, etc. Behaviour and electrophysiology, neural networks, multi-lead EEGs, non-invasive brain imaging technology, etc., are all hot topics among academics. Brain structure is best understood by looking at how the brain forms during development, a new study area and a major trend in neuroscience today. This overview focuses on three areas: EEG analysis, EEG acquisition, and EEG application. Li et al. presented four different EEG acquisition techniques, five EEG processing systems, and three application patterns. Electrode scalp placement, isolating amplification, digitising, and wireless transmission are the main components of an EEG acquisition device. The primary goals are low power usage, compact size, and portability [16]. Current research progress and future directions for suitable electrodes, a high integration acquisition chip, and an analysis method are discussed. Recent developments in EEG signal processing have enabled it to be used in settings outside its initial use in medical rehabilitation, including car safety, academic help, and even entertainment.

EEG is a biosignal that differs greatly from person to person due to its sensitivity and weakness. It is highly susceptible to distortions and background noise. Therefore, building an EEG acquisition system with signal integrity in mind is essential. Alkhorshid et al. suggested an analogue design for acquiring EEG signals [17]. The proposed design consists of eight blocks: (1) a radio-frequency interference filter and electro-static discharge protection, (2) a preamplifier and second-order high-pass filter with feedback topology and an unblocking mechanism, (3) a driven right leg circuit, (4) two-stage main and variable amplifiers, (5) an eight-order anti-aliasing filter (AAF), (6) a six-order 50 Hz notch filter (optional), (7) an optoisolator circuit, and (8) an isolated power supply. The design has the highest gain of about 94 dB and a bandwidth of about 0.18−120 Hz. The notch filter has a depth of −35 dB at 50 Hz. Because of issues with EEG integrity at frequencies between 40 and 60 Hz, its use is entirely discretionary.

The primary purpose of the paper by Jalagam and Mittal is to acquire EEG data, analyse it, and present the results [18]. A novel preprocessing technique using a high-impedance, high-CMRR amplifier is included. The system also includes anti-noise and anti-aliasing analogue filters. The MATLAB FFT method is used for the analysis. The presentation of EEG signals includes the discovery of the signals’ frequency corresponding to maximal magnitude. Due to their low frequency and amplitude, preprocessing, filtering, and converting biomedical signals accurately necessitate sufficient sampling rate, processing speed, gain, power consumption, and size.

The aim of Usakli’s research was to show some realistic state-of-the-art factors to consider when acquiring good signals for electroencephalographic signal acquisition. Users and system creators alike should keep these things in mind. Improving the system’s measurement performance depends on several factors, including the selection of appropriate electrodes and the design strategy of the original electronic circuitry front end [19]. Accuracy in biopotential measurements can be improved by keeping potential pitfalls in mind during system design and session recording. Electrode efficacy in EEGs is affected by system electronics (such as filtering, amplification, signal conversion, and data storage) and environmental factors. The principles of EEG electrodes and the main points of electronic noise reduction techniques in the front end of EEG signal acquisition are addressed in this work, along with some ideas for enhancing signal acquisition.

Many studies have been conducted in an attempt to explain human social contact because of its significance to the human condition. Some researchers have been trying to implement cutting-edge brain signal acquisition systems that track real-time neural activity during conversations. Even though multilateral contacts are the norm, most prior studies have only examined dyadic interactions. Because of methodological constraints, however, it is reportedly difficult to design a well-controlled experiment for multiple users at a reasonable cost. Most studies have instead concentrated on interactions between two users. As a result, multi-user devices for simultaneous acquisition are still a challenge. Lee et al. proposed a design framework for an acquisition device that can collect EEG data from a group of ten or more individuals in a single session [20]. Using the proposed framework, we collected EEG data from as many as 20 individuals at once at a frequency of up to 1 kHz. The hardware and software that comprise our acquisition system are explained in detail. Problems with synchronisation, system loads, electrodes, and applications are also addressed as they emerged during the system’s development. Nine participants took part in a series of simultaneous visual event related potentials experiments to further verify the efficacy of the suggested EEG acquisition framework. Our system performed admirably, with an average loss rate of only 1% and a delay of less than 4 ms. This method has potential applications in a wide range of hyper-scanning research, including studies of crowd behaviour, large-scale human interactions, and BCI collaboration.

After selecting the appropriate settings and electrode, the device can obtain real-time acquisition of EEG data, and the data can be displayed and saved, as described by Xingpan and Yang. It is highly accurate, compact, and energy-efficient [21]. The device is built around ST’s STM32F103VET6 microcontroller and TI’s ADS1299 analogue front-end IC, cutting out unnecessary peripheral circuitry. The source code for the application has been compiled with VC to receive EEG data and correctly show it in the display software. The acquisition device communicates with the host computer for data transfer via a wireless protocol. The research paves the way for BCI’s implementation in real-world settings.

In their short paper, Ren et al. described an analog front end (AFE) that can be used to identify EEG signals. The AFE has four parts: a chopper-stabilised amplifier, a ripple reduction circuit, a low-pass FIR filter based on random access memory, and an 8 bit SAR ADC [22]. For the first time, the bio-plausible characteristics of RRAM are used to efficiently evaluate signals in the analogue domain in an EEG AFE using a lowpass finite-impulse-response (FIR) filter. The symmetrical OTA design in the preamp minimises power usage while providing adequate gain. The noise characteristics and offset voltage are significantly enhanced by the ripple reduction circuit. A cutoff frequency of 40 Hz is achieved by the RRAM-based LPF, making it appropriate for the study of EEG data. The SAR ADC uses a segmented capacitor construction to reduce the energy used for capacitor switching. The test chip is built with 40 nm CMOS technology. Ultra-low-power operation is achieved with a total power usage of about 13 W [23 24 25].

## 3 The proposed method

### 3.1 Architecture for ADC

**Figure 1:**
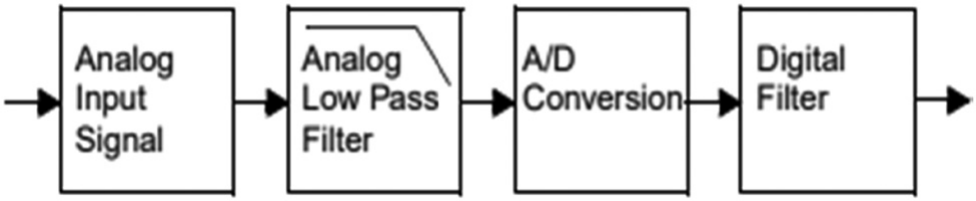
Process flow in data acquisition system.

The data acquisition comprises the AAF integrated with the CGR ADC model. The constructed model comprises the input analogue signal with the analogue LPF. The data acquisition system comprises the analogue-to-digital conversion followed by the digital filter. The complete process for the data acquisition scheme is presented in Figure 1.

The system comprises a DAQ signal for the sensor waveform with the application of the LPF or AAF. The operational amplifier (OP-AMP) model comprises the buffer configuration model. The output of the amplifier buffer comprises the resistor or capacitor pair to achieve the ADC input. The signal is processed with a successive approximation converter, and data acquisition is performed [26 27 28].

### 3.2 AAF

**Figure 2:**
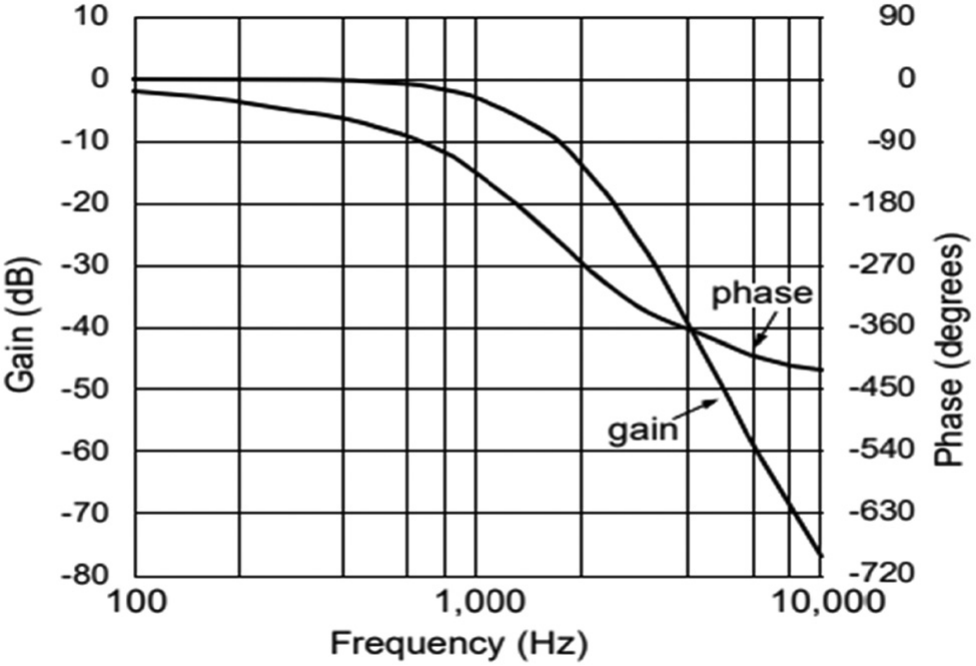
Frequency response of fifth-order Bessel design.

**Figure 3:**
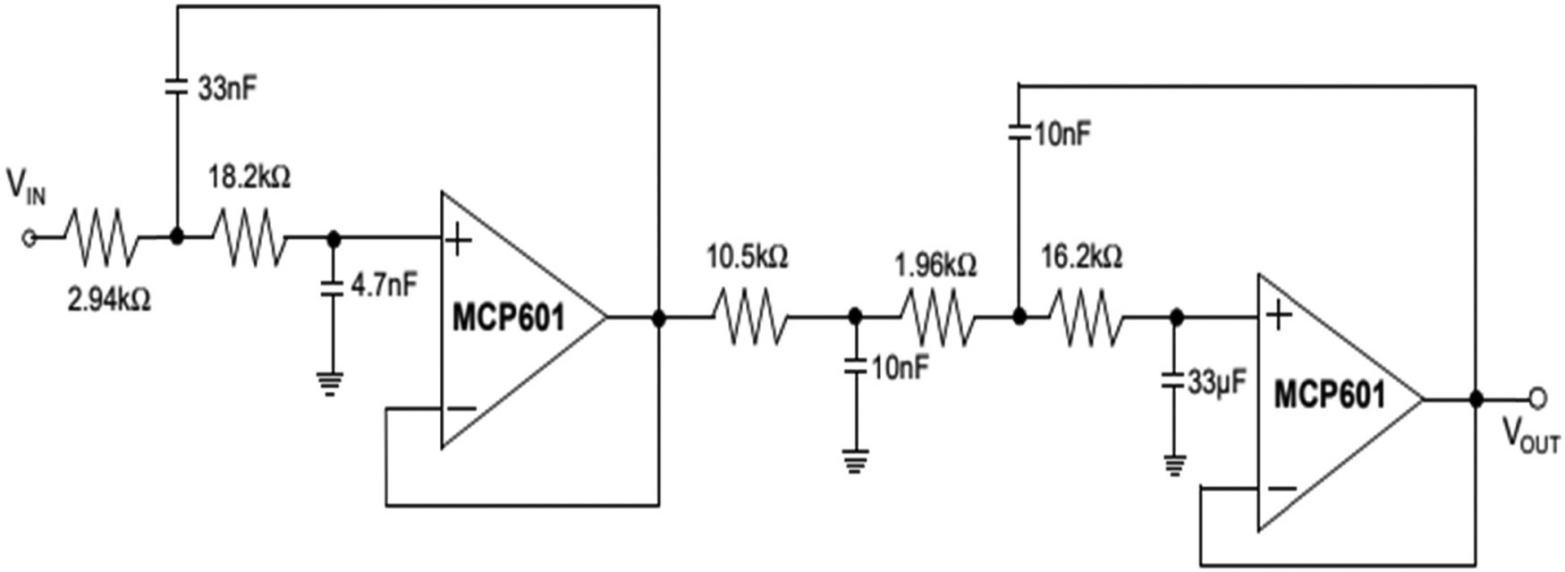
Fifth-order Bessel design implemented with two Sallen–Key filters using the passive filter.

The analogue signal forms an active LPF with the conversion of analogue signal bandwidth in the frequency of 1 kHz. The LPF eliminates the high-frequency signals and the aliasing errors in the ADC. Based on the design parameter, the implementation order of the filter is altered. The use of SAR ADC segments the LPF components with the 12- bit frequency of operation. The ADC segments the LPF with the 12-bit SAR to process the ADC. The sampling rate of the ADC is stated as 20 kHz, with a Nyquist rate of 10 kHz. The AAF’s signal-to-noise ratio is the 12-bit ADC with a frequency of 74 dB. Figure 2 illustrates the Bessel filter implemented with the AAF for data acquisition. The filter’s cutoff frequency is observed as the fifth-order, with a frequency of 1 kHz utilised for implementation. The circuit design for the Sallen–Key filter is combined with the passive LPF presented in Figure 3. The attenuation in the filter has an analogue input signal value of 79 dB with a pass band value of 10 kHz. The Bessel response frequency with the fifth-order filter is presented in Figure 2.

The Bessel filter is designed with the AAF with a cutoff frequency of 1 kHz and a stop band frequency of ∼5 kHz.

### 3.3 ADC design

The ADC model design estimates the data acquisition process in the EEG signal by eliminating the aliasing using low-pass Bessel filters. The design of the CGR ADC is presented as follows.

The pipelined *N*-stage ADC architecture operates simultaneous, consecutive samples *N* for the input analogue signal. Each pipelined ADC comprises the input voltage. Those are quantised with the high-resolution M-bit ADC to generate digital estimation, Vin. The estimation of digital coarse is converted into the form of a sub-digital-to-analogue converter (sub-DAC) eliminated from the input voltage for the factor gain of *G* = 2*M*. The pipelined architecture model comprises the backend stage-1, stage-3 and *N*. Each pipeline architecture estimates the redundancy to eliminate the residue for the clipping. Each stage output is integrated with the inverse gain for the estimation of analogue input as in equation (1).

(1) \[{D}_{{\mathrm{out}}}={D}_{1}+\frac{{D}_{2}}{{G}_{1}}+\frac{{D}_{3}}{{G}_{1}{G}_{2}}+\ldots \ldots \ldots \ldots \ldots .+\frac{{D}_{N+1}}{{G}_{1}{G}_{2}\ldots \ldots {G}_{N}}.\hspace{ 1em}]\]

Switched capacitor output characteristics for the M-bit stages are presented in equation (2).

(2) \[{V}_{{\mathrm{res}}}=\frac{\mathop{\sum }\limits_{i=1}^{{2}^{M}}{C}_{i}{V}_{{\mathrm{in}}}-\mathop{\sum }\limits_{i=1}^{{2}^{M}-1}{C}_{i}{T}_{i}{V}_{{\mathrm{REF}}}}{{C}_{{\mathrm{F}}}+\frac{{C}_{{\mathrm{F}}}+{C}_{{\mathrm{P}}}+\mathop{\sum }\limits_{i=1}^{{2}^{{\mathrm{M}}}}{C}_{i}}{A}},]\]

which can be rewritten as in equation (3).

(3) \[{V}_{{\mathrm{res}}}=\alpha {V}_{{\mathrm{in}}}+\beta {V}_{{\mathrm{REF}}},]\]

where

\[\alpha =\frac{\mathop{\sum }\limits_{i=1}^{{2}^{M}}{C}_{i}}{{C}_{{\mathrm{eq}}}}]\]

and

\[\beta =\frac{\mathop{\sum }\limits_{i=1}^{{2}^{M}-1}{C}_{i}{T}_{i}}{{C}_{{\mathrm{eq}}}}.]\]

The infinite precision residue is computed as in equation (4).

(4) \[{V}_{{\mathrm{in}}}=\frac{{V}_{{\mathrm{res}}}}{\alpha }-\delta {V}_{{\mathrm{REF}}}.]\]

The equivalent digital estimation is computed for the analogue input and is presented as in equation (5).

(5) \[{D}_{{\mathrm{in}}}=\frac{{D}_{{\mathrm{BE}}}}{\alpha }-\delta .]\]

The estimation output topology is presented as in equation (6).

(6) \[{V}_{{\mathrm{out}}}=\frac{({C}_{1}+{C}_{2}){V}_{{\mathrm{in}}}-k{V}_{{\mathrm{REF}}}{C}_{1}}{{C}_{2}+\delta }.]\]

The non-flip output topology is estimated as in equation (7).

(7) \[{V}_{{\mathrm{out}}}=\frac{{C}_{1}{V}_{{\mathrm{in}}}-k{V}_{{\mathrm{REF}}}{C}_{1}}{{C}_{2}+\delta }.]\]

The calibration is performed to estimate the mismatch in the capacitance-voltage topology.

**Figure 4:**
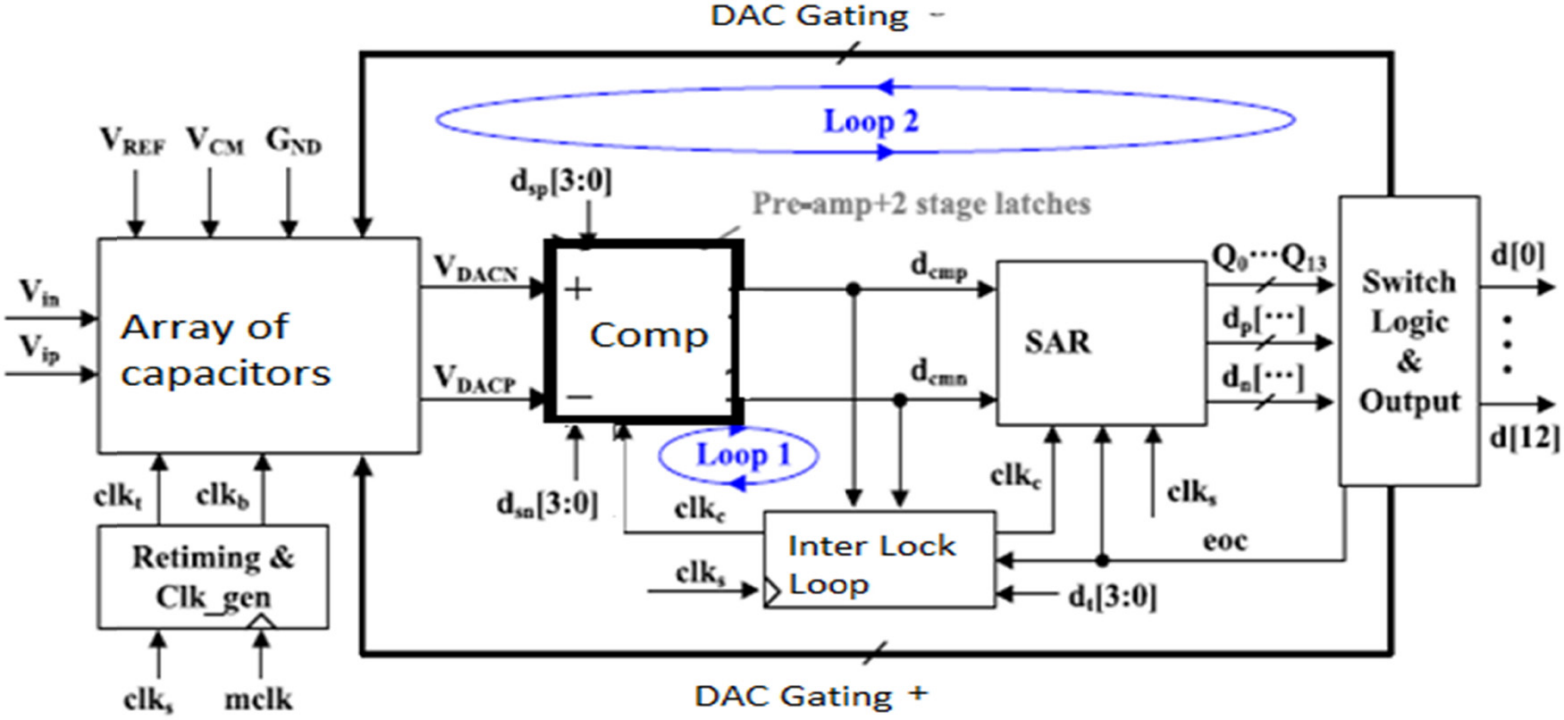
The proposed non-binary capacitor CGR ADC.

Figure 4 illustrates a 12-bit fully asynchronous differential process with the CGR ADC with the array of the non-binary capacitor. The configuration comprises the full differential improved with the rejection of the common-mode noises by doubling the signal voltage range and reducing the harmonic distortion. However, the key modules comprise CGR ADC design with the operation in low voltage for the DAC (non-binary and small capacitor unit) and comparator (preamplifier and latches in two stages). The asynchronous CGR ADCs comprise loop 1 and loop 2. The lowest loop determines the CGR ADC with the maximal speed value. Figure 4 comprises the CGR ADC with the Loop 2 delay with the main factor limits within ADC speed values. The high-speed CGR comprises a sampling rate of 100 MHz with a period of 10 ns generated with the multiphase clock generator with the master clock frequency of 1.2 GHz. The multi-phase clock circuit comprises the sample clock value of clks in ADC pulse value of 10/12:10 ns, with the sample pulse width of 833 ps. To eliminate the error, the pulse width is adjusted to 800 ps with the sampling signal reset of rst with low SAR logic and reset logic, settling DAC and the high set is stated as rst. The sample clock triggers comprise the falling edge with a comparison of a comparator, and subsequent cycle clocks are generated with a self-timed loop. The setup time of DAC comprises the adjustable delay cell with quantisation of Q13 varying from low to high end of conversion. The plate at the top is in reset state based on the next sampling signal values.

Although the flip-around topology is less attractive from a calibration perspective, it has two distinct advantages over the non-flip-around topology. First, the flip-around topology has a higher feedback factor and larger closed-loop bandwidth than the non-flip-around topology. Second, ignoring OP-AMP noise, the thermal noise contribution due to the sampling switches for both the flip-around and non-flip-around topology given in equations (8) and (9), respectively, clearly show that the non-flip-around topology is noisier.

(8) \[{N}_{{\mathrm{Th}},{\mathrm{f}}}=\frac{{kT}}{{C}_{1}+{C}_{2}}{\mathrm{\cdot }}{G}^{2},\hspace{ 1em}G={\mathrm{MDAC}}{\mathrm{gain}},]\]

(9) \[\hspace{1em}{N}_{{\mathrm{Th}},{\mathrm{nf}}}=\frac{{kT}}{{C}_{1}}{\mathrm{\cdot }}{G}^{2}+\frac{{kT}}{{{\mathrm{C}}}_{2}},\hspace{ 1em}G={\mathrm{MDAC}}{\mathrm{gain}}.]\]

The DAC capacitor mismatch and low OP-AMP DC gain are the major sources of distortion or non-linearity in the residue characteristics, degrading the pipelined ADC’s overall linearity. In order to mitigate the effects of low OP-AMP DC gain and mismatched capacitors, digital calibration techniques have been used to measure and correct the errors introduced by the same. Digital calibration is incredibly popular in the modern era, with the new deep sub-micron technologies offering faster, low-power, and area-effective digital circuits compared to older technologies.

Ideal residue characteristics for a 2-bit pipelined stage and how they are impacted by primary mistakes. Ideally, a 2-bit stage has input-output characteristics given by \[{V}_{{\mathrm{res}}}=2{V}_{{\mathrm{in}}}-\frac{j}{2}{V}_{{\mathrm{REF}}}]\] , where *j* = (−3,−1,1,3) denotes the height of the thermometer code. With capacitor mismatch and other non-idealities, the generalised equation can be written as in equation (10).

(10) \[{V}_{{\mathrm{res}}}=\alpha {V}_{{\mathrm{in}}}-\beta {V}_{{\mathrm{REF}}},]\]

where \[\alpha =\frac{{C}_{1}+{C}_{2}+{C}_{3}+{C}_{4}}{{C}_{{\mathrm{F}}}\left(1+\frac{{C}_{{\mathrm{F}}}+{C}_{{\mathrm{P}}}+\mathop{\sum }\limits_{i=1}^{4}{C}_{i}}{{{AC}}_{{\mathrm{F}}}}\right)}\hspace{1em}]\] and \[\beta =\frac{{C}_{1}+{C}_{2}+\ldots \ldots \ldots .+{C}_{j}}{{C}_{{\mathrm{F}}}\left(1+\frac{{C}_{{\mathrm{F}}}+{C}_{{\mathrm{P}}}+\mathop{\sum }\limits_{i=1}^{4}{C}_{i}}{{{AC}}_{{\mathrm{F}}}}\right)}]\] .

The ideal characteristic shows that the residue gain *α* is the same in all four regions, ideally equal to 2. However, if there is a capacitor mismatch between the capacitors *C*_1_ and *C*_4_ and with CF, accumulation of these mismatches in the numerator and other non-idealities like finite OP-AMP gain, input parasitic etc., in the denominator results in a residue gain not equal to 2. The mismatch between sampling capacitors also affects the linearity of the DAC, resulting in vertical jumps ∆*V* in the residue characteristics every time the output code is. According to capacitor mismatch, the vertical shift ∆*V* varies from region to region. During a transition from region *j* to region *j* + 1, the vertical jump ∆*V* is given in equations (11) and (12).

(11) \[\triangle V=({\beta }_{j+1}-{\beta }_{j}){V}_{{\mathrm{REF}}},\hspace{ 1em}]\]

(12) \[\hspace{2.3em}=\frac{{C}_{j}}{{C}_{{\mathrm{F}}}\left(1+\frac{{C}_{{\mathrm{F}}}+{C}_{{\mathrm{P}}}+\mathop{\sum }\limits_{i=1}^{4}{C}_{i}}{{{AC}}_{{\mathrm{F}}}}\right)}.]\]

Assuming that the residue is quantised by an infinite resolution backend ADC, we can say that the digital jump is equal to the analogue value ∆*V*. If there is a mismatch such as \[{C}_{j+1}=C+\triangle V]\] and the denominator is 2C, obtained as in equation (13).

(13) \[\triangle V=\left(1+\frac{\triangle C}{C}\right)\frac{{V}_{{\mathrm{REF}}}}{2},]\]

\[\left(\phantom{\rule[-0.75em]{}{0ex}},\frac{\triangle C}{C}\right)\frac{{V}_{{\mathrm{REF}}}}{2}]\] indicates the unwanted jump in the residue transfer curve. This vertical shift introduces missing codes in the overall ADC output and results in substantial harmonic distortion at the output. The effect of the gain error on the residue characteristic obtains the gain error computed as in equation (14).

(14) \[\triangle =\frac{1}{A\theta }.]\]

To eliminate the missing codes, we need a minimum OP-AMP DC gain which can be obtained by referring the gain error *∆* to the input and forcing the total input referred error to be less than the quantisation error of the ADC. If n-bits are resolved in the first stage, then the input referred gain error is given in equation (15).

(15) \[{\triangle }_{{\mathrm{in}}}=\frac{1}{A\theta {2}^{n}}.]\]

The input-referred error should be less than the quantisation error 1/2 *N* for an *N*-bit pipelined ADC. Assuming only gain error is present in the ADC as in equation (16).

(16) \[\frac{1}{A\theta {2}^{n}}\lt \frac{1}{{2}^{N}}.]\]

Hence,

\[A\gt \frac{{2}^{N-n}}{\theta }.]\]

### 3.4 Capacitor-mismatch calibration

The residue characteristics architecture model comprises of 2 bits with 2 bits/stage output voltage as presented in equation (17)

(17) \[{V}_{{\mathrm{out}}}=\frac{\mathop{\sum }\limits_{i=1}^{4}{C}_{i}{V}_{{\mathrm{in}}}-\mathop{\sum }\limits_{i=1}^{3}{T}_{i}{C}_{i}{V}_{{\mathrm{REF}}}}{{C}_{{\mathrm{F}}}+\eta },]\]

where \[\eta =\left({C}_{{\mathrm{F}}}+{C}_{{\mathrm{P}}}+\mathop{\sum }\limits_{i=1}^{3}{C}_{i}\right)/A]\] , *A* denoted the OP-AMP gain in finite range, \[{C}_{{\mathrm{P}}}]\] denoted the OP-AMP node input, and \[{T}_{i}]\] represents the flash ADC thermometer code. The digitised output voltage in the back end of ADC is presented in equation (18)

(18) \[{D}_{{\mathrm{BE}}}=\alpha {D}_{{\mathrm{in}}}-{\beta }_{i},]\]

where \[\alpha =\left(\mathop{\sum }\limits_{i=1}^{4}{C}_{i}\right)/({C}_{{\mathrm{F}}}+\eta )]\] denoted the gain in closed loop and \[{\beta }_{i}=\left(\mathop{\sum }\limits_{i=1}^{3}{T}_{i}{C}_{i}\right)/({C}_{{\mathrm{F}}}+\eta )]\] represented each node weights for the residue code.

**Figure 5:**
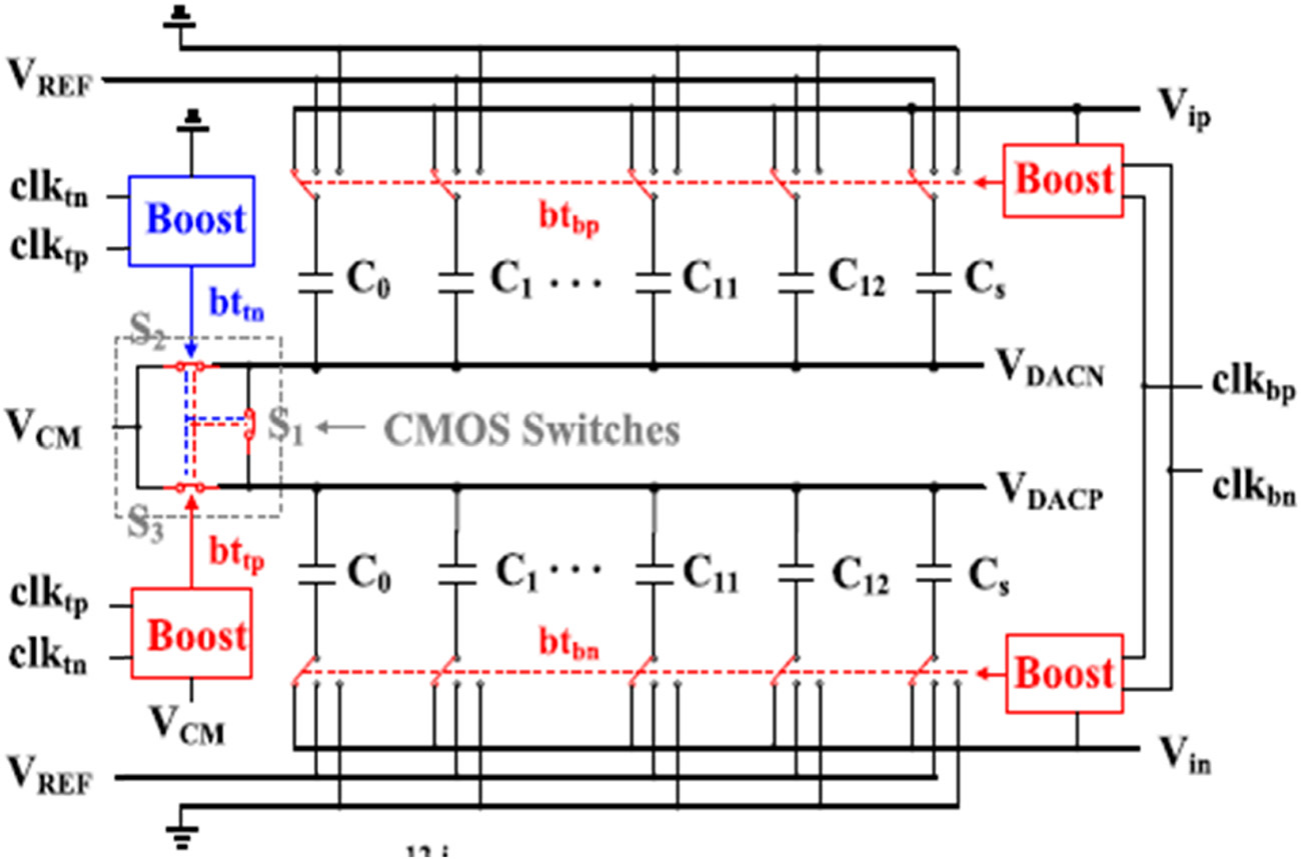
The proposed non-binary weighted capacitor array.

To achieve a high-accuracy sampling structure, the bottom plate is utilised as the switches in the bottom plate are controlled with the bootstrapped clock signal, as illustrated in Figure 5. The bootstrap circuit provides the minimal supply voltage with the small unit of the capacitor. The capacitor uses a pMOS/nMOS ratio (Wp/Lp:Wn/Ln) of 3:2 to incorporate charge injection and overcome the top plate leakage using CMOS switches in the top plate for lowering the injection charge and the clock feed. To reduce the leakage, the long channel length devices are implemented with the top plate switches controlled by a bootstrapped clock signal with the reduced switches in ON-resistance. Additionally, three switches are introduced with π-type, reducing ON-resistance to increase the VCM settling speed and accuracy.

The digital output goes from 0 to 3 as the input ranges from \[{-V}_{{\mathrm{REF}}}]\] to \[{+V}_{{\mathrm{REF}}}]\] . The redundancy in the circuit prevents the ADC from clipping. If threshold \[{V}_{{\mathrm{T}}0}]\] is applied to the stage under calibration and the comparator is forced to be in Regions 1 and 2, two digital outputs are obtained for the same input as in equations (19) and (20).

(19) \[{D}_{{\mathrm{in}}}{V}_{{\mathrm{T}}0}=\frac{{D}_{{\mathrm{BE}},0}^{{V}_{{\mathrm{T}}0}}}{\alpha }+\frac{{\beta }_{1}}{\alpha },\hspace{ 1em}]\]

(20) \[{D}_{{\mathrm{in}}}{V}_{{\mathrm{T}}0}=\frac{{D}_{{\mathrm{BE}},1}^{{V}_{{\mathrm{T}}0}}}{\alpha }+\frac{{\beta }_{2}}{\alpha }.\hspace{1em}]\]

Equating equations (19) and (20) gives the equation (21)

(21) \[{D}_{{\mathrm{BE}},1}^{{V}_{{\mathrm{T}}0}}-{D}_{{\mathrm{BE}},0}^{{V}_{{\mathrm{T}}0}}={\beta }_{1}-{\beta }_{2}.]\]

Similarly, the \[{\beta }_{i}-{\beta }_{i+1}]\] value in terms of the digital backend code for each threshold is given in equation (22).

(22) \[{D}_{{\mathrm{BE}},1}^{{V}_{{\mathrm{ri}}}}-{D}_{{\mathrm{BE}},0}^{{V}_{{\mathrm{Ti}}}}={\beta }_{i}-{\beta }_{i+1}.]\]

This provides equations (23)–(25).

(23) \[{D}_{{\mathrm{BE}}1,1}^{{V}_{{\mathrm{T}}1}}-{D}_{{\mathrm{BE}}1,0}^{{V}_{{\mathrm{r}}1}}={\beta }_{1}-{\beta }_{2},{\mathrm{\ }}]\]

(24) \[{D}_{{\mathrm{BE}}2,1}^{{V}_{{\mathrm{T}}2}}-{D}_{{\mathrm{BE}}2,0}^{{V}_{{\mathrm{r}}2}}={\beta }_{2}-{\beta }_{3},]\]

(25) \[{D}_{{\mathrm{BE}}3,1}^{{V}_{{\mathrm{T}}3}}-{D}_{{\mathrm{BE}}3,0}^{{V}_{{\mathrm{r}}3}}={\beta }_{3}-{\beta }_{1}.\hspace{1em}]\]

Implying the matrix form in equation (26)

(26) \[\left[\begin{array}{ccc}1 -1 0\\ 0 1 -1\\ 1 0 1\end{array}\right]\left[\begin{array}{c}{\beta }_{1}\\ {\beta }_{2}\\ {\beta }_{3}\end{array}\right]=\left[\begin{array}{c}{D}_{{\mathrm{BE}}1,1}^{{V}_{{\mathrm{T}}1}}-{{D}}_{{\mathrm{BE}}1,0}^{{V}_{{\mathrm{r}}1}}\\ {D}_{{\mathrm{BE}}2,1}^{{V}_{{\mathrm{T}}2}}-{{D}}_{{\mathrm{BE}}2,0}^{{V}_{{\mathrm{r}}2}}\\ {D}_{{\mathrm{BE}}3,1}^{{V}_{{\mathrm{T}}3}}-{{D}}_{{\mathrm{BE}}3,0}^{{V}_{{\mathrm{r}}3}}\end{array}\right].]\]

Thus, the capacitor mismatch coefficients *β*1–*β*4 compute the backend value *β* as shown below in equation (27).

(27) \[\left[\begin{array}{c}{\beta }_{1}\\ {\beta }_{2}\\ {\beta }_{3}\end{array}\right]=\frac{1}{2}\left[\begin{array}{ccc}1 1 1\\ -1 1 1\\ -1 -1 1\end{array}\right]\left[\begin{array}{c}{D}_{{\mathrm{BE}}1,1}^{{V}_{{\mathrm{T}}1}}-{D}_{{\mathrm{BE}}1,0}^{{V}_{{\mathrm{r}}1}}\\ {D}_{{\mathrm{BE}}2,1}^{{V}_{{\mathrm{T}}2}}-{D}_{{\mathrm{BE}}2,0}^{{V}_{{\mathrm{r}}2}}\\ {D}_{{\mathrm{BE}}3,1}^{{V}_{{\mathrm{T}}3}}-{D}_{{\mathrm{BE}}3,0}^{{V}_{{\mathrm{r}}3}}\end{array}\right].]\]

The capacitors *C*_1_ and *C*_4_ in by connecting *C*_4_ to ±*V*_REF_ controlled by thermometer code T0 and *C*_1_ to VCM. Instead of the *β*_1_–*β*_4_ values, we can obtain the new values defined as γ1–γ4. The new γ values for each region can calculate the gain as in equation (28).

(28) \[\alpha =-{\beta }_{1}+({\gamma }_{2}-{\gamma }_{1})/2.]\]

**Figure 6:**
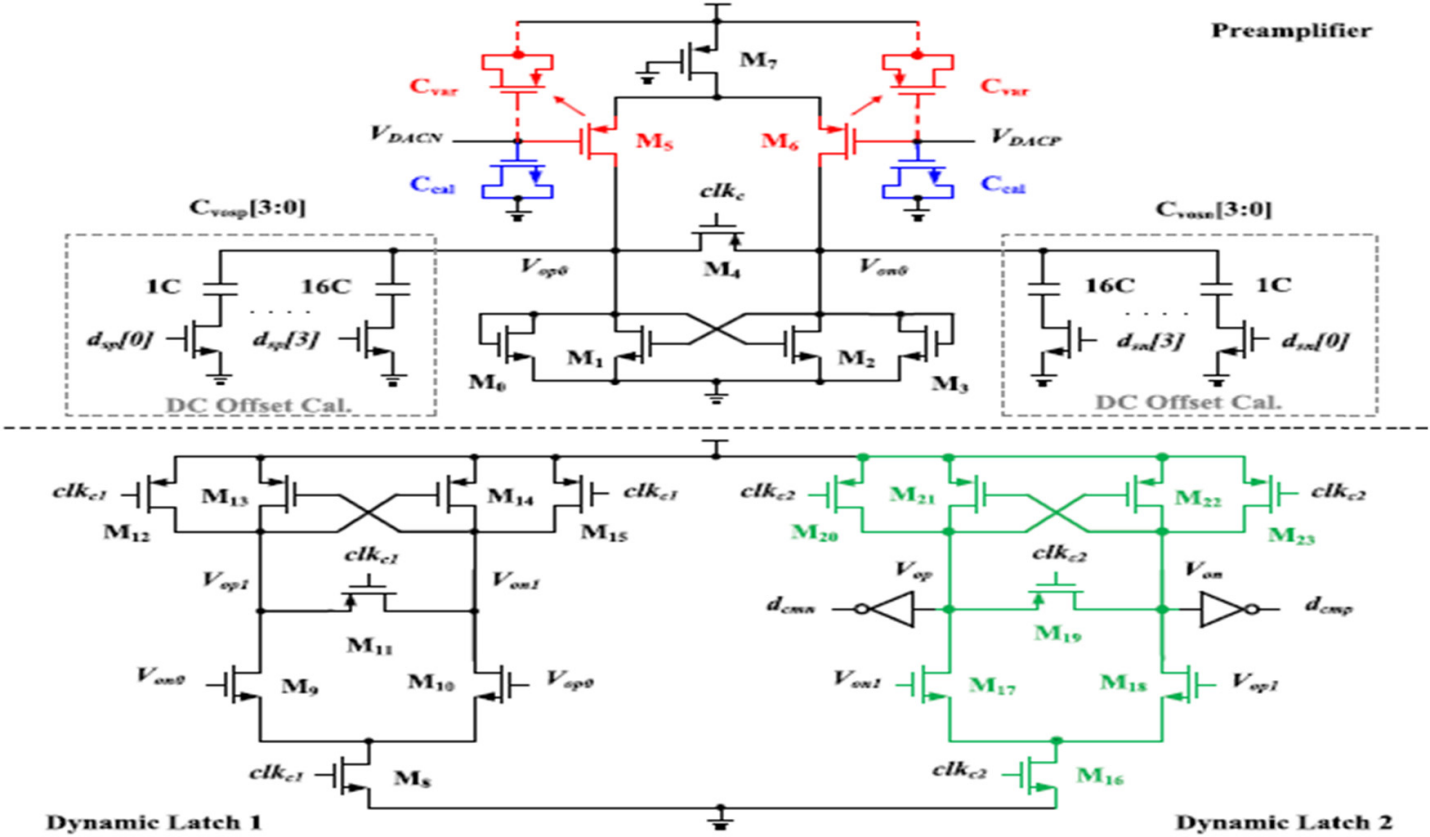
The proposed power optimised low-latency comparator.

The schematic of the low-latency comparator is presented in Figure 6, which are non-linear parasitic capacitor calibrations with the offset of 62 controllers with dsp[3:0] and dsn[3:0].

The capacitance unit is stated as 200 aF. The preamplifier output is equalised in the reset phase, and the preamplifier states M4. Adjustment for large-to-small signal swings caused by differential input voltage variations. Consider the data acquisition power consumption with the dynamic latch of the comparator. The circuit noises are minimised using input pair of p-type MOS. The ADC unit capacitor is at a shrinking level based on high speed and low power energy consumption for the parasitic capacitor at the input considered. The input pair of the parasitic capacitors Cvar for the capacitor MOS transistor is nonlinear.

## 4 Simulation results

**Figure 7:**
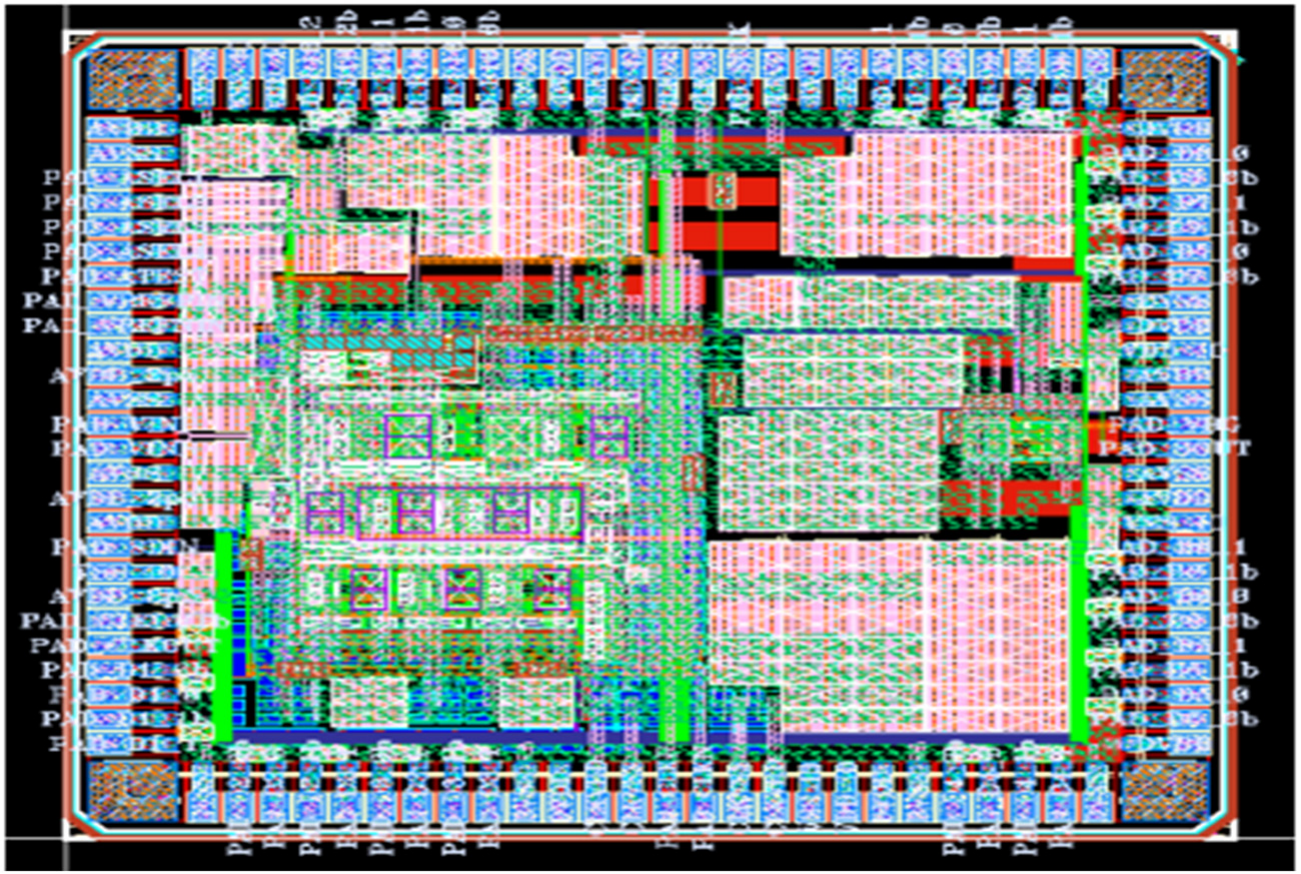
The layout of ADC.

The performance of the CGR is evaluated in the simulation software Spice for the computation of efficiency. To reduce the anti-aliasing effects for the EEG signal acquisition and amplification, non-binary CGR is implemented for the analysis. The OP-AMP design provides the 50 dB gain for the peak-to-peak linearity value of 75 dB with a swing value of 1.5 V with the UMC 65 nm process. The design comprises the 20 mA with a power supply of 1.5 V for the capacitor mismatch of 3% in the backend stage considered ideal. An effective number of bits (ENOB) of 11.8 bits is reported for the pipelined design of the ADC model. The nonlinearity estimation with the pipeline architecture for the bottom plate sampling with switches is focused on eliminating nonlinearity in the sampling switches, as presented in Figure 7.

Table 1 compares this ADC’s performance with prior ADCs with comparable resolution and sampling rates.

**Table 1:** Comparison of the performance of the ADC with that of prior art

	[2]	[29]	Proposed model
Technology (nm)	65 nm CMOS	90 nm CMOS	UMC 65 nm
Resolution (bits)	11	10	12
Conversion rate	1 GHz	500 MHz	500 MHz
SNDR (dB)	52.4	53	65.5
Power (mW)	33	55	103
Supply voltage (V)	1.2	1.2	1.2/2.5
Input voltage (V)	1.2*V*pp	1.2*V*pp	1.2*V*pp
Active ADC area	750 µm x 300 µm	0.5 mm × 0.5 mm	2.5 mm × 2.5 mm

The proposed model is implemented in the TANNER simulation software based on considering different conditions. Twelve bits of pipelined architecture are used for the simulation analysis, with three phases providing either four bits of resolution or three bits of resolution. Each stage’s redundancy value is measured as the 1 bit, and the inter-stage gain is 8 for the overall resolution value of 12 bits and 15 raw bits in ADC. The developed model comprises the calibration technique incorporating 2 states with the pipelined architecture for the backend process in an ideal state. Table 2 provides the key parameters incorporated in the two stages for the calibration of the noise circuit for every state with a resolution of 11.3 bits.

**Table 2:** Simulation Parameters

Stage no.	Gain of OP-AMP (%)	Comparator offset (mV)	Mismatch in capacitor (%)
1	100 ± 20	15	3
2	100 ± 20	15	3

The maximum code for the estimation incorporating counters and comparators with the simulation comprises the 9-bits with 10-bit counters with a maximum count value of 1,024. The obtained power is presented in Table 3.

**Table 3:** Power obtained for various subcircuits

Circuits simulated	Total power	Average power
Dynamic comparator	941.31 µW	806.6 nW
Sub-ADC	349.45 µW	3.136 µW
Sub-DAC	412.00 µW	177.3 µW
Stage 1	2.0327 mW	411.6 µW
OP-AMP sharing stage	1.887 mW	719.0 µW
Flash ADC	2.414 mW	55.39 µW
Final block	9.77 mW	4.748 µW

The conversion time of the 10-bit pipelined ADC can be calculated using equation (11)

Conversion time = 1/(clock frequency × No. of bits)

Clock frequency = 80 MHz, No. of bits = 10

Therefore, the conversion time obtained = 1.25 ns

The proposed pipelined ADC is implemented in 180 nm CMOS technology and occupies a die area of 1.3 mm². The measured DNL and the INL are +0.32/−0.32 LSB and +0.67/−0.67 LSB, respectively. Figure 7 shows the DNL and INL plots of 10-bit pipelined ADC, respectively. The measured SNDR for the input frequency of 2 MHz at 40MSPS is 55.67 dB, and ENOB is 8.95 and consumes 9.77 mW from 1.8 V. Figures 6 and 7 show the SNDR plot of a 10-bit pipelined ADC. Table 4 shows the power obtained for various subcircuits.

**Table 4:** Characteristics of OP-AMP

Reference	Technology (nm)	Supply Voltage (V)	Resolution (bits)	Sampling rate (MSPS)	SNDR (dB)	SFDR (dB)	ENOB (bits)	DNL/INL (LSB)	Power (mW)	Area (mm^2^)
[11]	180	1.8	10	30	58.50	66.10	−	0.30/0.46	21.6	1.85
[12]	180	1.8	10	30	57.41	65.93	9.10	0.57/0.80	21.6	0.70
[2]	28	2.0	12	30	52.5	66.70	8.6	0.37/0.87	11.0	0.94
[18]	90	2.1	14	16	61	79.2	9.57	+0.32/−0.28 + 0.62/−0.62	42.82	0.98
Present work	18	1.8	10	30	65.64	62.00	8.95	+0.32/−0.32/+0.67/−0.67	9.77	0.30

**Figure 8:**
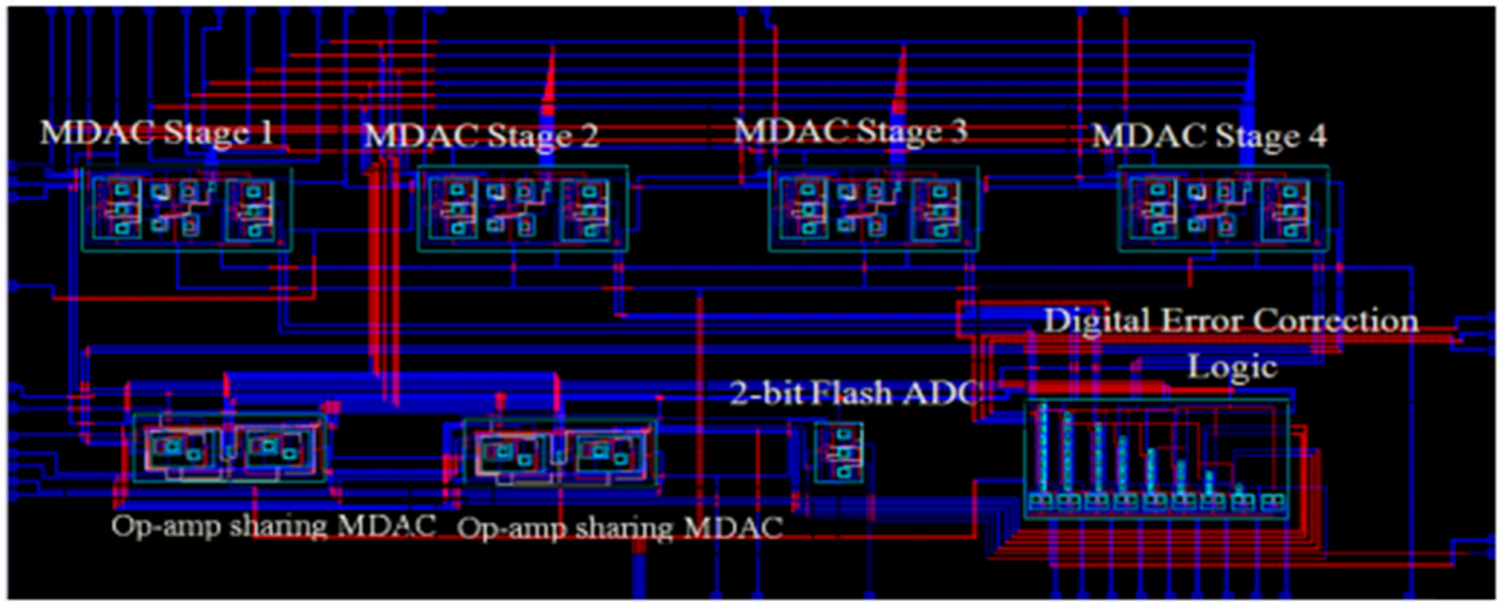
The layout of 10-bit pipelined ADC using MCS and OP-AMP sharing technique.

Figure 8 shows the layout of pipelined ADC. Various sub-blocks, including MDAC stage1, stage2, stage3, stage4, OP-AMP sharing MDAC, 4-bit flash ADC, and digital error correction logic, were integrated to form a 10-bit pipelined ADC.

**Figure 9:**
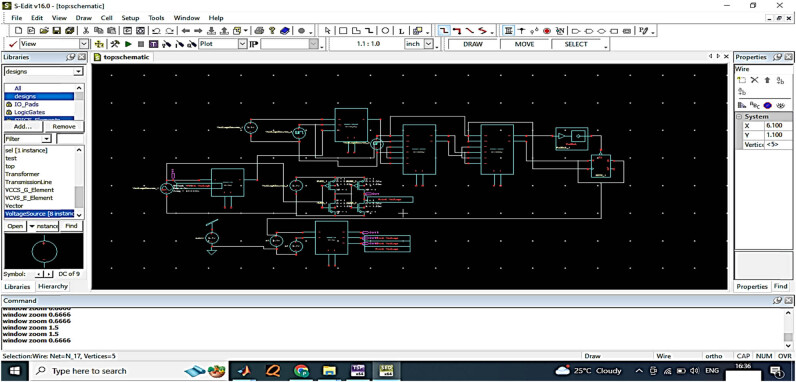
Top module schematic in S-edit.

Table 4 shows the performance specification of pipelined ADC. The CMOS design comprises the top and bottom plate comparators for eliminating anti-aliasing in the data acquisition process. Figure 9 provides the top module design of the comparators in the top view plate.

**Figure 10:**
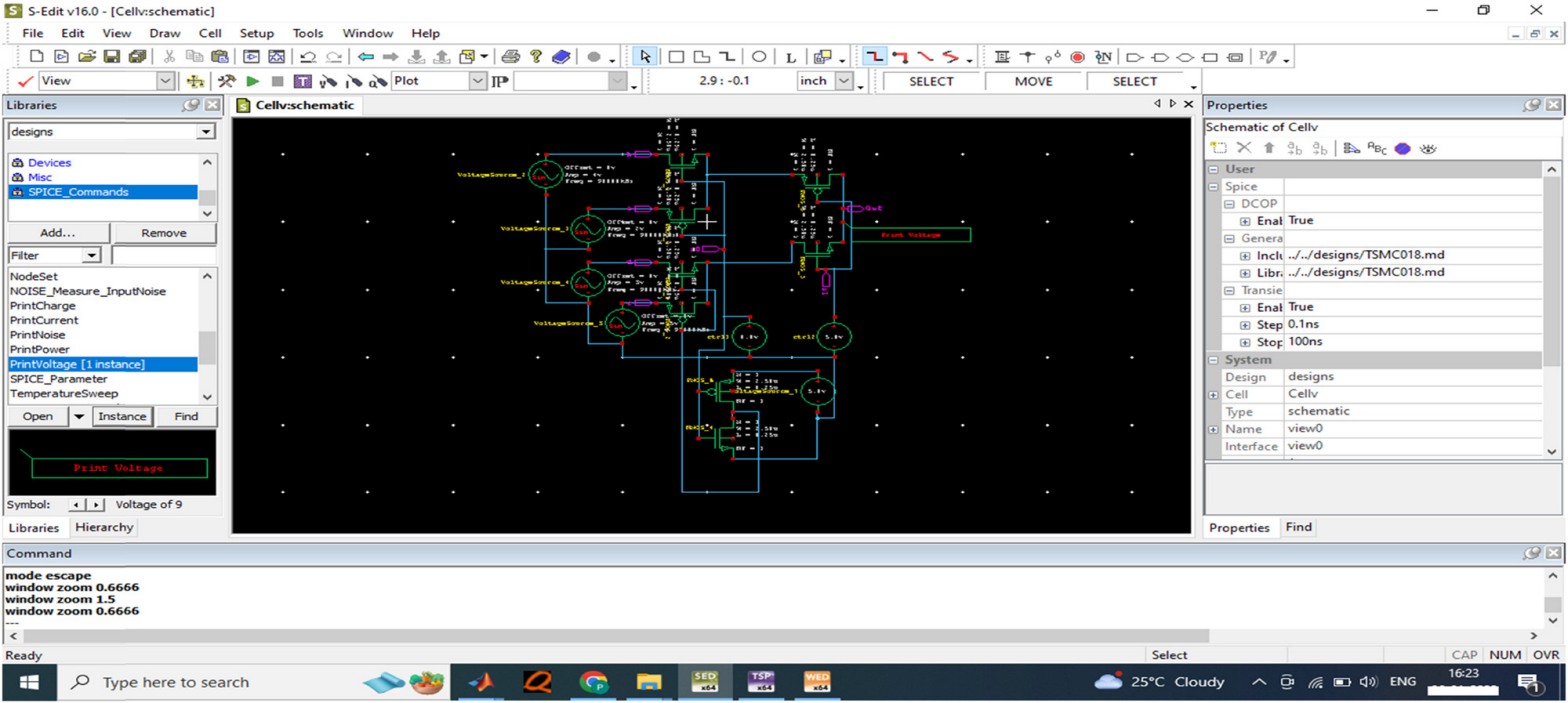
Tiny function generator design for input wave generation.

The data acquisition module is a critical component in generating and processing the EEG signal. With the design of the top module plate in the ADC design, the generator is designed with a tiny function, as shown in Figure 10, for generating the input waveform signal.

Based on the tiny function generator modules, the design computed the input wave generation for the varying levels of the voltages. The CGR ADC module estimates the voltages of 3 and 5 V, respectively.

**Figure 11:**
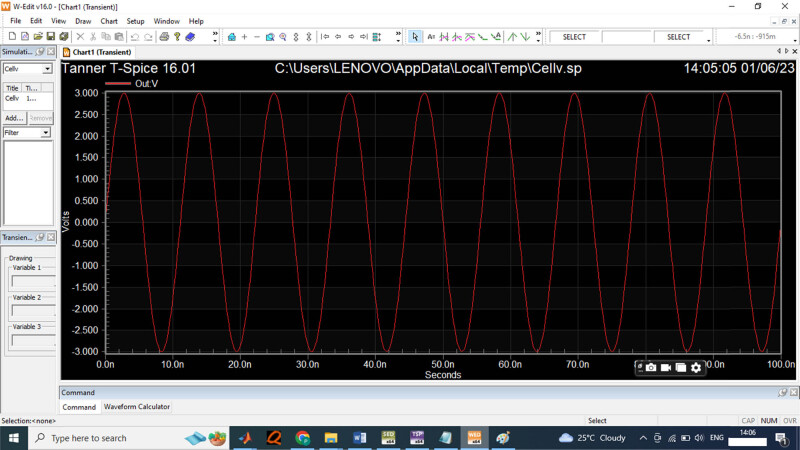
3 V input wave generated from function generator.

**Figure 12:**
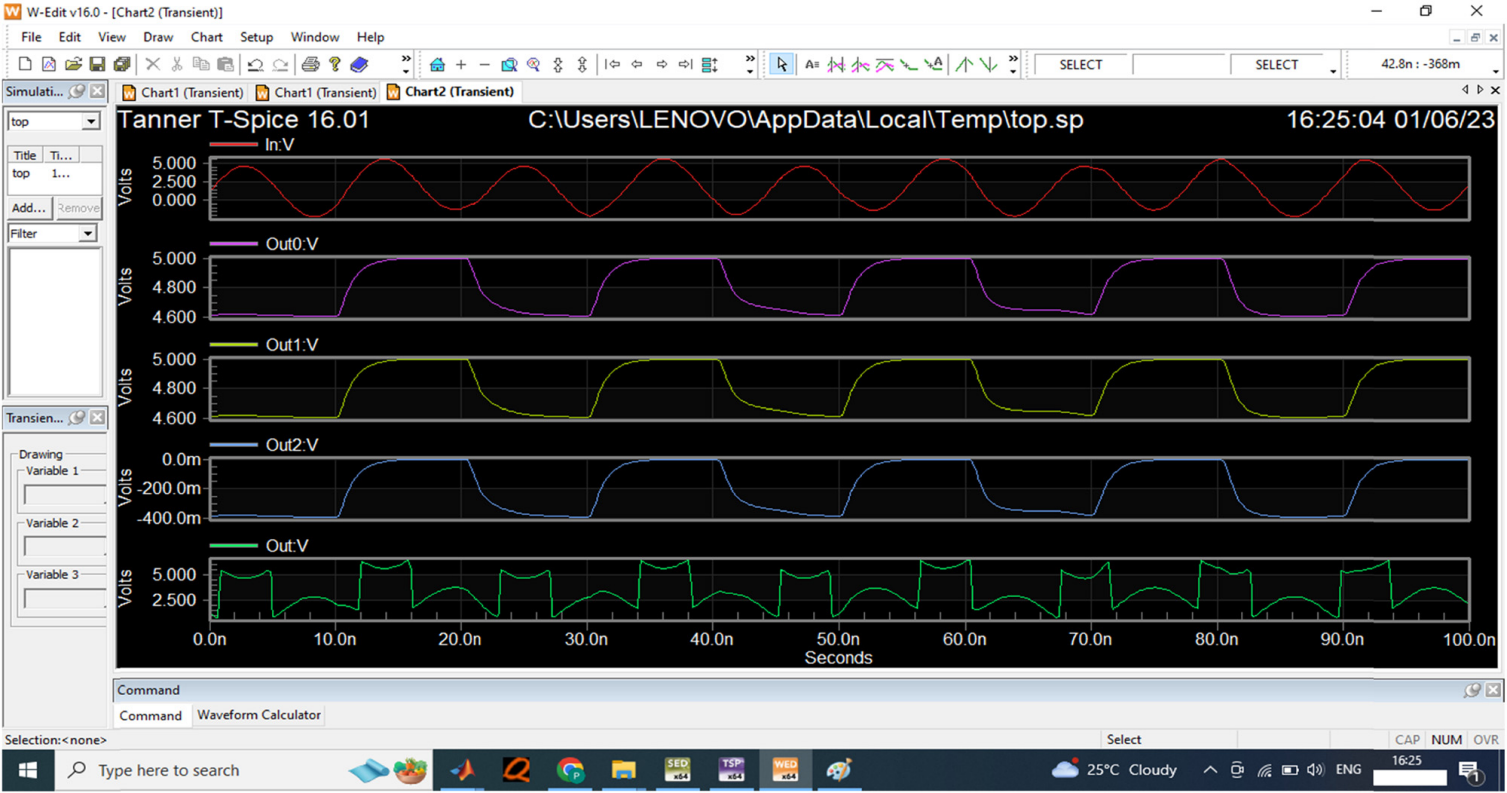
The proposed digital output for 3 V input sensor wave.

Figure 11 provides the function generator for the 3 V input voltage frequency for the data acquisition process. The output voltage measured for the 3 V input is computed as presented in Figure 12.

**Figure 13:**
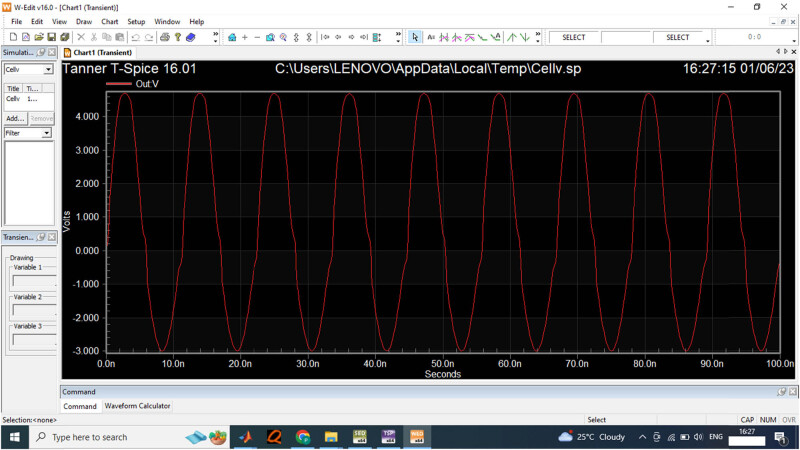
5 V input wave generated from function generator.

For the input sensor of 3 V, the performance of the designed ADC is evaluated in the Spice software, the time period measured is between 0 and 100 s for the variation in signal frequencies. The output voltage is measured as 3 V for the data acquisition process in the comparator. Figure 13 provides the 5 V input waveform for the data sensor to compute the signal frequencies.

**Figure 14:**
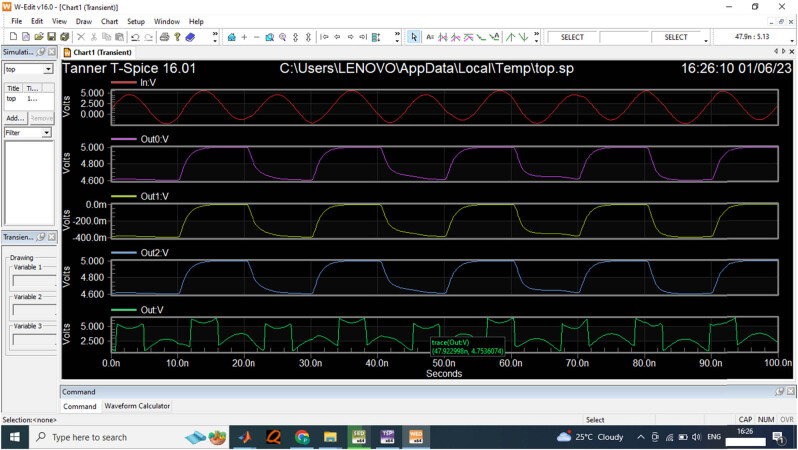
The proposed digital output for 5 V input sensor wave.

Figure 14 provides the input signal provided for the EEG sensor to estimate the output signal. The analysis expressed that the developed non-binary ADC generates the output value 5 V for the proposed ADC model. Table 5 compares the proposed ADC model with the non-linear capacitance, switch capacitor time, and power.

**Table 5:** Comparative analysis

	Power	Time (s)
Proposed	0.71365 nW	3.98
SAR ADC nonlinear capacitance	2.6 mW	10.5
IADC single-ended switch-capacitor	677 mW	32

### 4.1 Comparative discussion

The comparative analysis showed that the proposed model exhibits significant performance compared to the nonlinear capacitance and switch capacitor. The proposed model achieves a power consumption value of 0.713 nW, significantly less than the nonlinear and switch capacitors as those achieving a power consumption levels of 2.6 and 677 mW. Regarding time analysis, the proposed model exhibits a minimal processing time of 3.98 s, while the nonlinear capacitor achieves a time of 10.5 s and a switch capacitor value of 32 s.

## 5 Conclusion

Research on EEG covers data gathering, analysis, energy renewal, and more. EEG-gathering devices include encoding, digital transfer, head sensor placement, and separate amplifiers. The EEG detects periodic noise. Head movement, sensor lines, and hair sweat produce low-frequency noise. Low-frequency noise alters EEG signals over time. Muscle actions and electromagnetic waves create high-frequency noise. EEG shifts are saw-toothed by high-frequency noise. High- and low-frequency noises are usually lower and higher than human EEG, respectively. Lowering signal power above and below the testing level without altering the signs of interest lowers noise. Aliasing may affect low-frequency impacts in the original data because high-frequency noise is mirrored in the data. The SAR ADC system incorporates the filter evaluated based on the specification for the converter with DC- and AC-amplifier. The aliased signal path is considered with the elimination of the outside signal with sampling bandwidth in the ADC converter. The CGR ADC architecture utilises the high threshold for the voltage cells with the open loop comparator with an OP-AMP with high-voltage cells to minimise the power consumption and delay in CMOS. The DAC block comprises the resistor string designed with CMOS technology with higher threshold voltages. The constructed ADC techniques minimise the ADC power consumption and delay. The present study comprises the programmable ADC 1 A with low power to derive high efficiency from operating an appropriate field in ADC. The presented data acquisition scheme comprises the EEG signal monitoring for amplification with the digital codes of 8 or 12 cycles with the code size of 100kS/s/100 MS/s with a sampling rate. The adjustment unit in ADC comprises the reconfigured unit that is synchronised. The comparative analysis expressed that the proposed ADC model achieves the minimal energy consumption value of ∼12%, which is minimal than the nonlinear and switch end capacitor. Also, the time consumed is ∼9% less than the nonlinear and switch-end capacitor.
